# Diminished fibrotic encapsulation and comparable physicochemical properties of poly (styrene-*b*-isobutylene-*b*-styrene) support its use as a biomaterial alternative to silicone implants

**DOI:** 10.3389/fbioe.2026.1748501

**Published:** 2026-02-26

**Authors:** Nikita Kalashnikov, Leonard Pinchuk, Joshua Vorstenbosch

**Affiliations:** 1 Division of Surgical and Interventional Sciences, Department of Surgery, McGill University, Montreal, QC, Canada; 2 Research Institute of the McGill University Health Centre, Montreal, QC, Canada; 3 Faculty of Medicine and Health Sciences, McGill University, Montreal, QC, Canada; 4 Innovia, LLC, Miami, FL, United States; 5 Department of Biomedical Engineering, University of Miami, Miami, FL, United States; 6 Division of Plastic and Reconstructive Surgery, Department of Surgery, McGill University Health Centre, Montreal, QC, Canada

**Keywords:** foreign body response, PDMS, poly (styrene-block-isobutylene-block-styrene), SIBS, silicone

## Abstract

Silicone – namely polydimethylsiloxane (PDMS) – is a widely recognized elastomeric biomaterial commonly used in implantable medical devices such as breast implants, cardiac pacemakers and drug delivery devices. Despite its widespread use, PDMS can elicit a strong foreign body response with fibrous encapsulation that leads to discomfort, pain and implantable device failure in approximately 10% of cases. Poly (styrene-*block*-isobutylene-*block*-styrene) (SIBS) is a thermoplastic elastomer used clinically as a drug-eluting coating for coronary stents and experimentally in ocular drainage devices. Although SIBS has demonstrated excellent biocompatibility in these applications, the foreign body response it elicits has not yet been extensively studied in more inflammation-prone anatomic sites such as skin rich in macrophages and fibroblasts. Here, we characterize the physicochemical properties of SIBS, examine its effect on macrophage-fibroblast interactions and evaluate its biocompatibility by implanting it subcutaneously in mice to ultimately assess its viability as a potential alternative to PDMS. We establish that both materials have comparable physicochemical properties, demonstrate that fibroblasts adopt a less contractile pro-inflammatory phenotype when exposed to SIBS-macrophage conditioned media and show reduced fibrotic encapsulation around SIBS implants in mice. These results suggest that SIBS could potentially be a favorable biomaterial alternative to silicone in clinical applications.

## Introduction

Implantable medical devices reduce mortality and improve quality of life: breast implants restore the form of the breast, cardiac pacemakers modulate the heart’s electrical activity, cochlear implants provide a sense of hearing, hydrocephalus shunts drain excess cerebrospinal fluid and subdermal birth control implants deliver contraceptive medication. Although these devices restore the form and function of the human body as well as enable drug delivery, more than 10% of these devices fail over time (Zhang et al., 2021) as a result of the host’s response to the device’s biomaterial components that are in contact with tissue. This foreign body response occurs at the biomaterial-tissue interface where it ultimately produces a fibrous capsule that separates the host from the biomaterial.

The foreign body response (FBR) is typically characterized by protein adsorption, inflammation and fibrosis (Anderson et al., 2008; Kalashnikov et al., 2025). When a synthetic non-biodegradable biomaterial is implanted into the human body, proteins from blood and/or other bodily fluids first rapidly adsorb onto the biomaterial surface, with adsorption profiles varying based on the material’s surface properties (Rahmati and Mozafari, 2018; Chen et al., 2024). Then, immune cells infiltrate into the implantation site and sustain inflammation originally initiated by surgical trauma. Macrophages, in particular, not only clear the area by phagocytosing microbes and cellular debris, but they also adhere to the protein-coated biomaterial and respond to it with phenotype polarization, which allows them to reversibly acquire a specific phenotype with a defined secretome of regulatory cytokines. For example, classically-activated pro-inflammatory M1 macrophages release IL-1β and IL-6, whereas alternatively-activated immunoregulatory or wound-healing M2 macrophages secrete IL-10 and TGF-β (Sheikh et al., 2015; Liu and Segura, 2020; Kalashnikov and Moraes, 2022). Depending on their activation state, macrophages can utilize these cytokines to recruit and direct the behavior of other cells such as lymphocytes to establish chronic inflammation (Adusei et al., 2021) and/or connective tissue cells to initiate tissue repair (Witherel et al., 2019). Notably, fibroblasts are ultimately responsible for fibrosis around the biomaterial by laying down extracellular matrix (ECM) proteins like collagen and remodeling the resulting matrix into a fibrous capsule especially in response to stimulation by macrophages with TGF-β, which turns them into contractile myofibroblasts (Witherel et al., 2019; Noskovicova et al., 2021a). Ideally, the foreign body response ends with minimal fibrous encapsulation, but sometimes it can become dysregulated and result in a thick vascular contractile fibrous capsule.

Silicones exist in various forms ranging from oils to rubbers, but all share the same fundamental polymeric siloxane backbone. The simplest and most widely used silicone is polydimethylsiloxane (PDMS) characterized by having pendant methyl groups. It is a synthetic thermoset elastomer, which is often transparent, quite flexible and hydrophobic. Since PDMS lacks reactive functional groups and can withstand relatively harsh physicochemical conditions, it has become a widely recognized biomaterial used in many implantable medical devices (Quinn and Courtney, 1988; Zare et al., 2021). For example, it is used as a bulk material for breast implants and hydrocephalus shunts, or as a coating for pacemaker leads and cochlear implant electrode arrays. Furthermore, it is also utilized as a subdermal drug delivery vehicle for hormonal contraceptive medication. However, PDMS can elicit a strong foreign body response with significant clinical consequences: capsular contracture due to excessive fibrous encapsulation occurs in approximately 20% of women with breast implants (Shin et al., 2018; Foroushani et al., 2022; Gorgy et al., 2023) and around 40% of hydrocephalus shunts obstruct within 2 years of implantation presumably due to inflammatory tissue infiltration (Bigio and R, 1998; Harris and McAllister, 2012). Not only do these medical device failures cause pain and/or progression of the underlying condition, but they almost always require revision surgery, which further contributes to implant-related patient morbidity and burdens the healthcare system with additional costs. Moreover, the use of PDMS might limit the potential benefits of implantable medical devices made out of it. For instance, fibrous encapsulation of PDMS-based cochlear implants and subdermal contraceptive implants can affect the performance and/or lifetime of these devices by respectively increasing cochlear electrode impedances (Foggia et al., 2019) and hindering drug delivery (Fayzullin et al., 2021). Hence, there is a clear need to find biomaterial alternatives to PDMS that elicit a milder foreign body response with minimal fibrous encapsulation.

Poly (styrene-*block*-isobutylene-*block*-styrene) (SIBS), on the other hand, is a synthetic thermoplastic elastomer consisting of a self-assembled physically-crosslinked network of linear triblock copolymer styrene-isobutylene-styrene chains (Pinchuk et al., 2008). Given that it does not feature cleavable side groups, SIBS is thought to be resistant to oxidation, hydrolysis and enzymatic degradation making it exceptionally biodurable (Pinchuk et al., 2008). This has led it to be successfully used clinically as a paclitaxel-eluting coating for coronary artery stents (TAXUS^®^ stent) (Kamath et al., 2006) and experimentally in ocular shunt devices (Pinchuk et al., 2017). In the former application, coronary artery restenosis rates are reduced from 27% to 8% compared to bare metal stents at 9 months (Stone et al., 2004), while – in the latter – no clinically significant inflammation or fibrous encapsulation is observed around ocular SIBS shunt devices (Pinchuk et al., 2016). However, the eye is considered an immune-privileged anatomic site where immune-mediated inflammation is naturally suppressed (Streilein, 2003; Niederkorn, 2006) and the cardiovascular system – more specifically, the heart valves (Hill et al., 2021) and the arterial tunica media (Tellides and Pober, 2015) – also appears to exhibit some immune privilege. This indicates that the foreign body response is likely different in these locations compared to other sites, yet there are no rigorous studies on the foreign body response to SIBS in more conventional inflammation-prone anatomic sites such as skin. In this work, we aim to evaluate the foreign body response associated with SIBS and determine if SIBS can be a suitable biomaterial alternative to PDMS. To do so, we first characterize the physicochemical properties of SIBS, then we examine how it affects macrophage-fibroblast phenotypes *in vitro* and finally we implant SIBS subcutaneously in mice, all while comparing it to PDMS.

## Materials and methods

Unless otherwise stated, all cell culture materials and supplies were purchased from Fischer Scientific (Ottawa, ON), and all chemicals were acquired from Sigma-Aldrich (Oakville, ON). PDMS was prepared in-house using a Sylgard 184 silicone elastomer kit (No. 4019862; Dow), while medical-grade 1 mm thick SIBS sheets (InnFocus, Inc.) were supplied directly by LP. Human monocytes (THP-1; TIB-202; ATCC) were generously donated by Dr. Lisbet Haglund (McGill University). Human primary dermal fibroblast extraction, expansion and usage was approved by the MUHC Research Ethics Board (MP-37-2020-5995). 8-week old C57BL/6J mice were obtained from the internal colony of the Animal Resources Division (ARD) at the Research Institute of the MUHC (RI-MUHC) and all mice experiments were approved by the Animal Care Committee of the RI-MUHC (MUHC-10152) in accordance with the Canadian Council on Animal Care (CCAC) guidelines.

### Biomaterial preparation

PDMS was prepared by mixing the polymer base with the curing agent in a 10:1 mass ratio, degassing the resulting mixture, pouring it into a Petri dish and curing it in a convection oven at 70 °C for 24 h. The prolonged curing time was selected to help stiffen the PDMS and render it more hydrophilic to better match SIBS without making it excessively brittle (Seghir and Arscott, 2015). For tensile tests, a 3D-printed polylactic acid (PLA) ASTM D638 IV dumbbell mold was utilized; otherwise, PDMS was biopsy punched into 10 mm disks for *in vitro* use or into 4 mm disks for animal experiments. SIBS sheets were directly carefully cut into the same shapes using a scalpel or biopsy punches. To ensure sterility in experiments with cells and mice, both biomaterials were soaked in 70% ethanol for 5 min and washed with PBS three times, and then exposed to UV for 1 h. For cell culture, the 10 mm biomaterial disks were also glued to the bottom of 12-well plates beforehand by using drops of uncrosslinked PDMS and placing the resulting constructs into the oven for 30 min.

### Tensile testing

To characterize the mechanical behavior of both biomaterials, tensile testing was conducted using a CellScale UniVert machine equipped with a 200 N load cell. Briefly, the thickness, width and length of the gauge sections of the dumbbell biomaterial samples were measured with a micrometer. Then, each sample was aligned along the loading axis, securely mounted in the machine and stretched at a constant rate of 10 mm/min until breakage. The recorded force-displacement data and the calculated original cross-sectional area of the samples was used to generate engineering stress-strain curves. The Young’s moduli were obtained from the linear region of the stress-strain curves, which corresponded roughly to the initial 0.1% strain range, using linear regression.

### Contact profilometry

A Bruker Dektak profilometer armed with a 2 μm diamond-tipped stylus was utilized to study surface topography. The stylus was traversed across the biomaterial surface over a scan length of 2 mm for a duration of 30 s with a 3 mg force. Surface profile data was recorded, filtered using Gaussian regression with a 0.25 mm long cutoff and analyzed to quantify relevant surface roughness parameters (e.g., R_a_ for average roughness, R_q_ for root mean square roughness, R_sk_ for surface profile skewness and R_ku_ for surface profile kurtosis) in Vision64. Three measurements were taken from different regions of each sample to account for surface heterogeneity.

### Contact angle goniometry

To assess surface wettability, static water contact angle measurements were performed using the sessile drop method with a DataPhysics OCA 15EC contact angle goniometer. 2 μL of deionized water was gently deposited at a rate of 0.5 μL/s on the biomaterial surface using a micro-syringe and images of the resulting droplets were captured immediately. Using the SCA20 software, droplet profile extraction was performed and contact angle values were computed with the Young-Laplace fitting algorithm. For each sample, three measurements were taken in different locations. In addition, all measurements were obtained in one session without interruption to ensure consistent ambient temperature and humidity conditions.

### Cell culture

THP-1 human monocyte cultures were kept in Roswell Park Memorial Institute 1,640 medium (RPMI 1640; Gibco) supplemented with 10% fetal bovine serum (FBS), 1% antibiotic-antimycotic (AA), 1% 0.2 mM (w/v) L-glutamine in PBS and 0.05 mM β-mercaptoethanol at 37 °C and 5% CO_2_ in suspension T-25 flasks. Cell counts and trypan blue live-dead assays were regularly performed to ensure an adequate cell concentration of 0.2–0.8 million cells per mL with a viability of at least 90%. Cultures were maintained either by adding fresh complete RPMI 1640 media, starting a new flask or resuspending the entire culture in media after centrifuging it at 200 g for 5 min. For experiments, THP-1 monocytes between passage 3 and 8 were recovered by centrifugation, resuspended in complete RPMI 1640 media with 25 nM phorbol 12-myristate-13 acetate (PMA) at a density of 0.15 million cells per mL, added to the biomaterials and left to differentiate into macrophages over 48 h (Figure 2A) as previously done elsewhere (Kalashnikov and Moraes, 2023). The biomaterial surfaces were then washed with pre-warmed PBS to remove unattached poorly-differentiated THP-1 cells and, after adding fresh media, the cultures were left to rest for 1 day (Figure 2A). Then, 72 h later, macrophage cultures were imaged and cellular RNA was extracted at which point conditioned media was also collected (Figure 2A).

Human primary dermal fibroblasts were grown in Dulbecco’s modified Eagle’s medium (DMEM; Gibco) supplemented with 10% FBS and 1% AA at 37 °C and 5% CO_2_ in T-25 flasks. Cultures were trypsinized and passaged into new flasks when they reached 75% confluency. For experiments, fibroblasts from passages 5 to 8 were harvested with trypsin and either seeded at a density of 0.0125 million cells per mL into well plates or used for collagen gel contraction assays at a concentration of 1 million cells per mL (Figure 3A). In both cases, they were subjected to a 1:1 mixture of macrophage-conditioned media and fresh complete DMEM media (Figure 3A) where the conditioned media was first spun at 1,000 rpm for 15 min to remove any cellular debris as done previously (Chen et al., 2023).

### Microscopy and image analysis

An EVOS M5000 microscope with 10x and 20x magnifications was used to capture images of cell cultures as well as stained fibrous capsule tissue sections, which were then both analyzed using ImageJ (NIH). For the former, all cells were counted in at least three fields of view for each well and freehand outlined as well as had their area and Feret diameters measured. Aspect ratio was calculated by dividing the maximum by the minimum Feret diameter. For the latter, the fibrous capsule thickness was measured in at least three locations in each capsule where it was intact with no visible gaps due to possible sectioning artifacts. Cell density was calculated by counting the number of cells within at least three 1,000 μm^2^ regions in at least three fibrous capsule samples.

### Gene expression analysis

In order to study macrophage and fibroblast phenotypes, total RNA was first extracted from cell cultures using the RNeasy Fibrous Tissue Mini Kit (Qiagen) according to the manufacturer’s instructions. RNA concentration and purity were evaluated using the NanoDrop 2000 UV-Vis spectrophotometer (Thermo Fisher Scientific). Then, cDNA was synthesized using the GoScript Reverse Transcription oligo (dT) Mix (Promega) from 500 to 1,000 ng of total RNA following the manufacturer’s recipe and recommended thermal cycler parameters. Finally, gene expression was assessed using quantitative polymerase chain reaction (qPCR) performed on an Applied Biosystems StepOnePlus machine with SYBR Green chemistry according to the recommended instructions. Primer sequences used for qPCR can be found in Table 1. Relative gene expression levels were obtained from three technical replicates, and calculated using the ΔΔC_T_ method (Livak and Schmittgen, 2001; Taylor et al., 2019) where the housekeeping GAPDH gene and the TCPS condition were used for normalization.

**TABLE 1:** RNA primer sequences used for qPCR.

Gene	Forward primer sequence	Reverse primer sequence
*GAPDH*	5′-TCC​CTG​AGC​TGA​ACG​GGA​AG-3′	5′-GGA​GGA​GTG​GGT​GTC​GCT​GT-3′
*NOS2*	5′-GTT​CTC​AAG​GCA​CAG​GTC​TC-3′	5′-GCA​GGT​CAC​TTA​TGT​CAC​TTA​TC-3′
*IRF5*	5′-ACC​TCA​GCC​CTA​CAA​GAT​CTA​CGA-3′	5′-AAC​ATC​CTC​TGC​AGC​TCT​TCC​TC-3′
*ARG1*	5′-GAC​AGA​CTA​GGA​ATT​GGC​AAG​GTG-3′	5′-GGG​TCC​AGT​CCG​TCA​ACA​TCA​AAA-3′
*STAT6*	5′-ATC​ACC​ATT​GCC​CAT​GTC​ATC​C-3′	5′-ACC​CAT​CTG​TTC​AGG​CTT​GTA​GT-3′
*IL1B*	5′-GAC​AAG​CTG​AGG​AAG​ATG​CT-3′	5′-AGC​GTG​CAG​TTC​AGT​GAT​CG-3′
*IL6*	5′-TGA​ACC​TTC​CAA​AGA​TGG​CTG-3′	5′-CAA​ACT​CCA​AAA​GAC​CAG​TGA​TG-3′
*CXCL8*	5′-CCT​TCC​TGA​TTT​CTG​CAG​CT-3′	5′-TGG​GGT​GGA​AAG​GTT​TGG​AG-3′
*TNF*	5′-TGA​CAA​GCC​TGT​AGC​CCA​TG-3′	5′-TTG​AAG​AGG​ACC​TGG​GAG​TA-3′
*TFGB1*	5′-GCA​CGT​GGA​GCT​GTA​CCA​GA-3′	5′-CGC​ACA​ACT​CCG​GTG​GAC​ATC-3′
*IL10*	5′-GGG​GAA​GGT​GAA​GGC​TCA​ATC​AAA-3′	5′-TCT​CGA​AGC​ATG​TTA​GGC​AGG​TT-3′
*COL1A1*	5′-GCT​TCA​CCT​ACA​GCG​TCA​CT-3′	5′-GAG​GGA​GTT​TAC​AGG​AAG​CA-3′
*COL3A1*	5′-TGC​CCC​AAC​CCA​GAA​ATT​CC-3′	5′-GGA​ATA​CCA​GGG​TCA​CCA​TT-3′
*ACTA2*	5′-CCG​GGA​GAA​AAT​GAC​TCA​AA-3′	5′-TAG​ATG​GGG​ACA​TTG​TGG​GT-3′

### Collagen gel contraction assay

To assess fibroblast contractility, human primary dermal fibroblasts were embedded in collagen gels as previously done elsewhere (Dubois et al., 2019; Kalashnikov and Moraes, 2019). Briefly, a neutralized collagen-cell mixture was prepared according to the recipe presented in Table 2 on ice and left to polymerize in 48-well plates inside of an incubator for roughly an hour. Then, a syringe needle was used to release the resulting collagen gels from the well edges in order to initiate free contraction, and a 1:1 mixture of macrophage-conditioned media and fresh complete DMEM media was added to the wells. Finally, the contracted gels were imaged 72 h later and contraction extent was quantified as a percentage by dividing the change in gel area at 3 days by the original area. To evaluate whether cell number affected gel contraction, the contracted gels were also digested with collagenase and total DNA was extracted using the DNeasy Blood & Tissue Kit (Qiagen) according to the manufacturer’s instructions. DNA content was then quantified with the NanoDrop 2000 UV-Vis spectrophotometer (Thermo Fisher Scientific).

**TABLE 2:** Collagen gel recipe.

Component	Volume (μL)
10X DMEM	20
1M sodium hydroxide (NaOH)	4.5
Sterile water	15.5
7.5% sodium bicarbonate (NaHCO_3_) buffer	10
1X DMEM	50
3 mg/mL bovine collagen type I (VWR)	150
Cell suspension	50
Final 1.5 mg/mL collagen gel volume	300

### Biomaterial implantation surgery

PDMS and SIBS disks were implanted under the dorsal skin in mice in order to evaluate the foreign body response to both biomaterials. First, the mice were given slow release buprenorphine (1 mg/kg) and carprofen (20 mg/kg) analgesia, and then anesthetized using isoflurane inhalation (3% in 1.5 L/min oxygen) in an induction chamber. While maintaining isoflurane anesthesia (2% in 1 L/min oxygen) using a nosecone system, the dorsum was shaved with an electric shaver, and disinfected with three alternating scrubs of 2% chlorhexidine and 70% ethanol. Then, a roughly 1 cm incision was made on the bulge at the midline in the dorsal skin to ensure reproducible surgical site healing (Yampolsky et al., 2024) (Figure 4A). Using blunt dissection, two lateral subcutaneous pockets were created on each side of the incision and irrigated with saline. After delivering a few drops of lidocaine-bupivacaine solution for local analgesia, two PDMS and two SIBS disks were inserted into the resulting pockets away from the midline (Figure 4A). Finally, the incision was closed using simple interrupted stitches with 4-0 polypropylene sutures. Surgery was performed on a heating pad, ophthalmic ointment was applied as needed and isotonic fluids (25 mL/kg) were administered. All mice were monitored post-operatively until full recovery, returned to their cages and received carprofen analgesia daily for 3 days. Mice were housed in an animal facility inside ventilated cages in groups of three to five under standard conditions with a 12-h light/dark cycle, controlled temperature and humidity, and *ad libitum* access to food and water.

### Fibrous capsule tissue histology

At either postoperative day 7 or 28, mice were euthanized with carbon dioxide asphyxiation under isoflurane anesthesia followed by confirmation via thoracotomy. Following euthanasia, the incision was reopened and the skin was reflected along with the encapsulated biomaterial disks. Then, the implants with surrounding fibrous capsule tissue were carefully excised along with adjacent skin and immediately fixed in 10% neutral-buffered formalin for at least 48 h at 4 °C until processing and embedding in paraffin. Embedded tissues were sectioned using a Leica microtome at 5 μm thickness, and stained with hematoxylin and eosin (H&E) as well as Masson’s trichrome (MT).

### Statistical analysis

All biomaterial characterization tests were performed on more than three material samples and all biological experiments included at least three biological replicates. Data were analyzed in Microsoft Excel using two-sided unpaired or paired t-tests and one-way analysis of variance (ANOVA) as applicable with statistical significance set at p-value <0.05.

## Results and discussion

### SIBS and PDMS have comparable physicochemical properties

**FIGURE 1:**
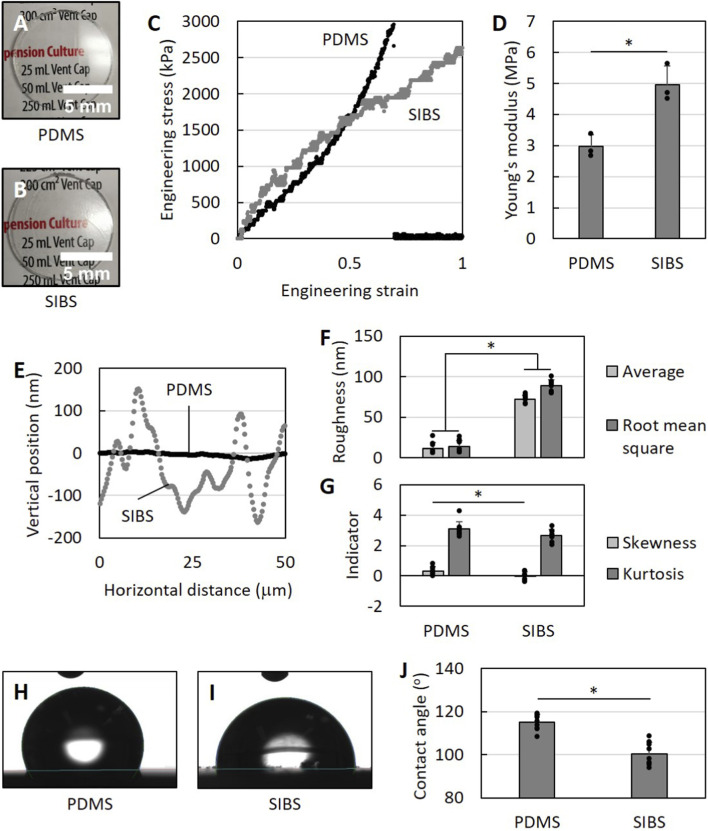
Physicochemical characterization of PDMS and SIBS. **(A,B)** Photographs of **(A)** PDMS and **(B)** SIBS disks. **(C)** Representative stress-strain curves for PDMS and SIBS, and **(D)** corresponding Young’s moduli that are within the same order of magnitude. **(E)** Example surface profiles, and **(F,G)** associated surface topography parameters for PDMS and SIBS with roughness values in the same 10-100 nm range. **(H,I)** Representative photographs of water droplets on **(H)** PDMS and **(I)** SIBS, and **(J)** matching contact angle values that are above 90°. Despite some differences, PDMS and SIBS are visually similar and have comparable physicochemical properties. Data reported as mean +/- standard deviation.

Since both biomaterials look virtually the same by eye (Figures 1A,B), we first compared their physicochemical properties. We conducted tensile testing (Figures 1C,D), contact profilometry (Figures 1E–G) and contact angle goniometry (Figures 1H–J) to characterize the stiffness, topography and wettability of both biomaterials.

We observed in the tensile testing experiments that SIBS behaves differently under load compared to PDMS. While PDMS is characterized by a nonlinearly elastic stress-strain relationship with strain-stiffening, SIBS displays strain-softening behavior (Figure 1C). Indeed, although SIBS is initially stiffer than PDMS, it becomes softer when its strain extends beyond 0.25 at which point the slopes of the stress-strain curves of both materials are roughly the same. This difference may be attributed to crosslinking, as SIBS is physically crosslinked with Van der Waals forces where interactions weaken with increasing strains as opposed to the much stronger covalent bonds that hold the chemically crosslinked PDMS together. The Young’s moduli are also significantly different with 2.98 ± 0.37 MPa for PDMS and 4.97 ± 0.60 MPa for SIBS (p-value = 0.004; Figure 1D), which is consistent with literature on PDMS with extended curing time (Seghir and Arscott, 2015) and SIBS (Ovcharenko et al., 2019). Nevertheless, the stiffness of these biomaterials is within the same order of magnitude.

Upon careful inspection, it appears that SIBS does not reflect light as well as PDMS (Figures 1A,B), suggesting different surface topography between the materials. In fact, contact profilometry shows that the surface profile of PDMS is much flatter than that of SIBS, which has prominent peaks and valleys (Figure 1E). The surface roughness parameters echo this observation: both average and root mean square roughness values are 72.3 ± 1.5 nm and 89.0 ± 2.4 nm for SIBS as expected (Yuan et al., 2014a) compared to 11.3 ± 4.3 nm and 13.5 ± 4.7 nm for PDMS (p-values <10^−5^; Figure 1F). The skewness of the PDMS surface profile is also significantly higher than that of SIBS indicating that the PDMS surface has more valleys (p-value = 0.017), while its kurtosis is not significantly different at an approximate value of 3 suggesting that the surface features of both biomaterials are neither too flat nor sharp (Figure 1G). Despite these differences, the scale of the topographical features of both biomaterials is in the same 10-100 nm range.

In terms of wettability, water droplets tend to be more spherical on PDMS (Figures 1H,I), indicating that PDMS is more hydrophobic than SIBS. Using contact angle goniometry, the contact angle is measured at 115.1 ± 3.8° for PDMS and 100.3 ± 5.4° for SIBS (p-value <10^−5^; Figure 1J) in accordance with SIBS literature (Ovcharenko et al., 2019; Yuan et al., 2014a; Yuan et al., 2016a). Although the contact angles are significantly different, both biomaterials are still considered hydrophobic, as their contact angles are above 90°.

### Macrophages express more pro-inflammatory genes on SIBS

**FIGURE 2:**
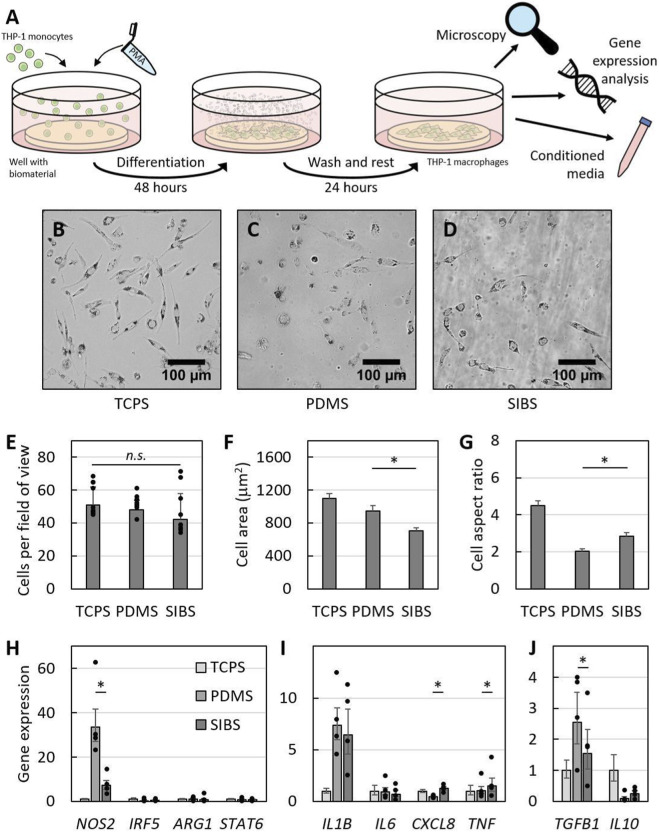
Macrophage phenotype on SIBS compared to PDMS. **(A)** Experimental diagram, demonstrating THP-1 monocyte differentiation into macrophages with 25 nM phorbol 12-myristate-13 acetate (PMA). **(B–D)** Representative microscope images of macrophages cultured on **(B)** tissue culture polystyrene (TCPS), **(C)** PDMS and **(D)** SIBS. **(E)** Macrophage attachment, **(F)** spread area and **(G)** elongation on the three different substrates, demonstrating that macrophages are smaller and more elongated on SIBS. **(H–J)** Macrophage expression of **(H)** M1 and M2 polarization markers as well as **(I)** pro-inflammatory and **(J)** anti-inflammatory genes across all three materials, indicating that macrophages adopt a slightly more pro-inflammatory phenotype on SIBS compared to PDMS. Data reported as mean +/- standard deviation except for **(F)** and **(G)** where standard error is used with n = 75–100 per material.

After demonstrating that PDMS and SIBS are comparable as materials, we then questioned if they would affect macrophages differently. We seeded THP-1 monocytes on both biomaterials, differentiated them into macrophages with 25 nM PMA for 48 h and let them rest for another 24 h to minimize inflammatory gene upregulation as recommended (Lund et al., 2016) (Figure 2A). Macrophage morphology was studied using microscopy (Figures 2B–G), and expression of key macrophage polarization markers (*NOS2*, *IRF5*, *ARG1* and *STAT6*) as well as inflammatory (*IL1B*, *IL6*, *CXCL8* and *TNF*) and anti-inflammatory (*TGFB1* and *IL10*) genes was evaluated using reverse transcription quantitative polymerase chain reaction (RT-qPCR) (Figures 2H–J).

Even though it was clear that macrophages look different on tissue culture polystyrene (TCPS) (Figure 2B) compared to PDMS (Figure 2C) and SIBS (Figure 2D), the number of cells per field of view across all three substrates was not significantly different (Figure 2E), indicating that macrophages attach equally well to both biomaterials and to a similar extent as to TCPS. This suggests that observed morphological changes are not due to cell attachment differences but are rather representations of differences in macrophage phenotype. Interestingly, macrophages are smaller and more elongated on SIBS compared to PDMS, as evidenced by a significantly lower cell spread area (p-value = 0.001; Figure 2F) and a higher cell aspect ratio (p-value = 0.003; Figure 2G). Although the link between THP-1 macrophage morphology and phenotype has not been fully established yet, literature suggests that cell spread area is associated with activation and elongated morphology corresponds to pro-inflammatory M1 macrophage polarization (Kalashnikov and Moraes, 2023; Zh et al., 2015). Given these correlations, SIBS might be perhaps polarizing macrophages more towards an M1 phenotype but generally activating less macrophages compared to PDMS although this remains speculative.

In terms of gene expression, only the expression of *NOS2* out of the two M1 polarization markers (*NOS2* and *IRF5*) is significantly different between both biomaterials with macrophages on SIBS transcribing less of it (p-value = 0.042; Figure 2H). Since there are no significant differences for M2 polarization markers (*ARG1* and *STAT6*), this suggests that macrophages on SIBS are less polarized towards an M1 phenotype. Seemingly in contradiction, these macrophages also have a significantly higher expression of pro-inflammatory *CXCL8* and *TNF* genes (p-values = 0.013 and 0.042; Figure 2I), and a significantly lower expression of the pro-fibrotic *TGFB1* gene (p-value = 0.037; Figure 2J). That being said, a single marker cannot accurately define macrophage polarization and, as such, we believe that macrophages cultured on SIBS might be polarized towards a slightly more pro-inflammatory phenotype compared to those on PDMS given their more elongated morphology and their higher expression of pro-inflammatory genes. However, more functional macrophage assays such as measurements of secreted cytokines are needed to further clarify the phenotype of THP-1 macrophages cultured on SIBS. Furthermore, although THP-1 macrophages were used due to ease of culture and availability while being human in origin, they may not accurately capture phenotype polarization shifts compared to primary macrophage cells (Chanput et al., 2014; Tedesco et al., 2018), which could potentially resolve the apparent contradictions in gene expression and should be used in future studies aiming to confirm the preliminary observations made here.

### Fibroblasts express pro-inflammatory genes and are less contractile when exposed to conditioned media from macrophages cultured on SIBS

**FIGURE 3:**
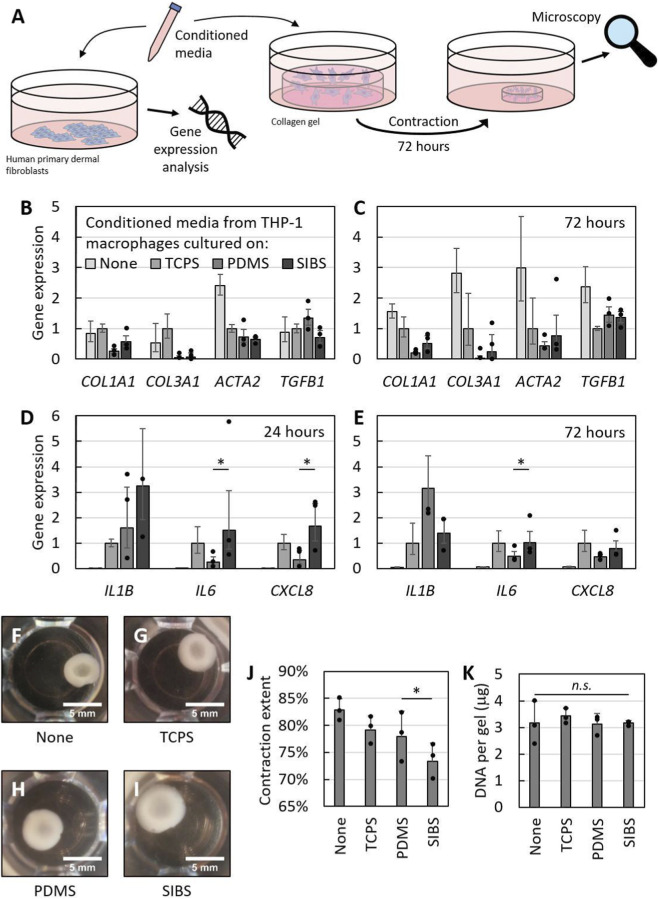
Fibroblast phenotype when exposed to conditioned media from macrophages cultured on TCPS, PDMS and SIBS. **(A)** Experimental diagram, illustrating how macrophage conditioned media was used to interrogate fibroblast phenotype. **(B–E)** Fibroblast expression of **(B,C)** ECM-related genes and **(D,E)** pro-inflammatory genes at **(B,D)** 24 h and at **(C,E)** 72 h, highlighting upregulation of pro-inflammatory genes in the SIBS condition. **(F–I)** Representative photographs of contracted fibroblast-laden collagen gels at 72 h after being subjected to **(F)** unaltered fresh media or conditioned media from macrophages cultured on **(G)** TCPS, **(H)** PDMS and **(I)** SIBS. **(J)** Contraction extent for the various gels at 72 h, demonstrating reduced fibroblast contractility in the SIBS condition. **(K)** Amount of DNA extracted from contracted fibroblast-laden collagen gels, indicating indirectly that all gels have the same amount of cells and proliferation is not a confounding factor for the observed contractility changes. Data reported as mean +/- standard deviation.

Given the altered phenotype of macrophages cultured on SIBS, we next assessed whether these macrophages could, in turn, influence fibroblast behavior. To study this, we added media conditioned by macrophages grown on both biomaterials to fibroblasts cultured on standard tissue culture plastic as per previous work (Chen et al., 2023) and to fibroblast-laden collagen gels (Figure 3A) in order to respectively evaluate their gene expression and contractility.

Since macrophages on SIBS had a higher expression of *TNF* as well as a lower expression of *TGFB1* than those on PDMS, we expected their conditioned media to downregulate collagen (*COL1A1* and *COL3A1*) and alpha smooth muscle actin (α-SMA encoded by *ACTA2*) genes, as TNF-α (Goldberg et al., 2007; Zhu et al., 2021) and TGF-β (Li et al., 2006; Finnson et al., 2013) work antagonistically to affect ECM production and myofibroblast differentiation characterized by high α-SMA levels. Surprisingly however, the expression of *COL1A1*, *COL3A1* and *ACTA2* is not significantly different at both 24 h (Figure 3B) and 72 h (Figure 3C) between the PDMS and SIBS conditions. Instead, pro-inflammatory *IL6* and *CXCL8* genes are significantly upregulated in fibroblasts exposed to SIBS-macrophage conditioned media at 24 h (p-values = 0.016 and 0.035; Figure 3D) and this is maintained until 72 h for *IL6* (p-value = 0.019; Figure 3E). Given the growing evidence that fibroblast activation is not restricted to myofibroblast differentiation and can result in adoption of a secretory or immune-interacting phenotype (Davidson et al., 2021; Schuster et al., 2021), we hypothesize that fibroblasts exposed to conditioned media from macrophages cultured on SIBS acquire an inflammatory phenotype characterized by secretion of inflammatory cytokines as opposed to a contractile or tissue-remodeling one.

To confirm whether the fibroblasts in the SIBS condition are shifting away from a contractile phenotype, we decided to look at their level of contractility using collagen gel contraction assays (Figures 3F–I). Indeed, fibroblasts exposed to conditioned media from macrophages cultured on SIBS contracted the collagen gels significantly less than those in the PDMS condition (p-value = 0.028; Figure 3J). To rule out proliferation as a potential confounder, we also digested the contracted collagen gels and quantified the amount of DNA in them as an indirect measure of cell number. There were no significant differences in amount of DNA extracted (Figure 3K), indicating that the fibroblasts in the SIBS condition are indeed less contractile. This is consistent with previous findings that interleukin-8 (IL-8 encoded by *CXCL8*) (Iocono et al., 2000; Ghazarian et al., 2000) and TNF-α (Goldberg et al., 2007; Zhu et al., 2021) suppress fibroblast contractility. Further work with protein assays is however required to fully tease apart the phenotype of fibroblasts exposed to conditioned media from THP-1 macrophages grown on PDMS and SIBS, as these fibroblasts might actually be adopting a mixed phenotype with myofibroblast (e.g., retained expression of *ACTA2*, *COL1A1* and *COL3A1* genes) and secretory (e.g., acquired expression of *IL6* and *CXCL8* genes as well as reduced contractility) characteristics.

### Fibrotic encapsulation around SIBS implants is diminished in mice

**FIGURE 4:**
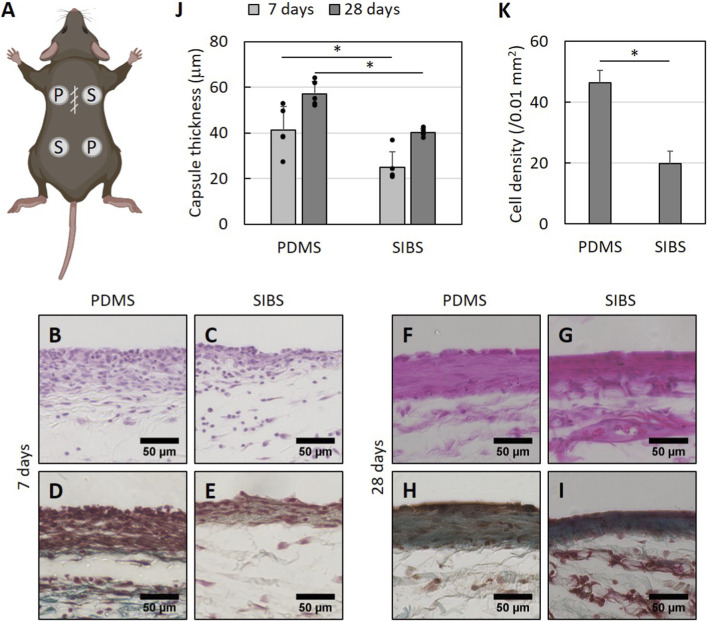
Foreign body response in mice to PDMS and SIBS. **(A)** Illustration of a mouse post-biomaterial implantation surgery, emphasizing the closed incision on the bulge of the dorsum and the four implant locations. **(B–I)** Representative microscope images of **(B,C,F,G)** hematoxylin & eosin **(H,E)** and **(D,E,H,I)** Masson’s trichrome stained fibrous capsule tissue sections harvested **(B–E)** 7 days or **(F–I)** 28 days postoperatively. **(J)** Fibrous capsule thickness around PDMS and SIBS implants at 7 days and 28 days, demonstrating reduced fibrous encapsulation around SIBS. **(K)** Cell density at 28 days, indicating a reduction in the amount cells around SIBS implants. Data reported as mean +/- standard deviation except for **(K)** where standard error is used with n = 9 per material.

To see if these *in vitro* observations translate to substantial differences between PDMS and SIBS in eliciting a foreign body response *in vivo*, PDMS and SIBS disks were implanted subcutaneously in mice (Figure 4A). The resulting encapsulated implants were harvested either 7 days or 28 days later and the capsular tissue was analyzed histologically. At 7 days, the fibrous capsules around both biomaterials were highly cellular with minimal collagen deposition indicative of the initial phase of the foreign body response (Figures 4B–E), while fibrous capsules at 28 days were more consistent with later stages of the foreign body response, being thicker, less cellular and rich in collagen (Figures 4F–I). For both time points, fibrous encapsulation around SIBS implants is significantly reduced compared to PDMS (p-values = 0.017 and 0.0002; Figure 4J). Moreover, the cell density of the fibrous capsules is significantly lower for SIBS (p-value = 0.0002; Figure 4K), indicating that the capsular tissue is less active. Along with more advanced *in vitro* macrophage-fibroblast direct co-culture experiments with more comprehensive readouts, immunohistochemistry staining for macrophage polarization and extracellular matrix-related markers is however necessary to understand the inflammatory and fibrotic profile of these fibrous capsules as well as the mechanism resulting in our findings.

Nevertheless, the findings presented here suggest that, in contrast to PDMS, SIBS elicits a milder foreign body response. Previously, unmodified SIBS has only been shown to achieve the same level of tissue reactivity as silicone under rabbit conjunctiva (Nakamura et al., 2022) within the immune privileged environment of the eye. Hence, our work not only extends this observation to skin but also demonstrates that SIBS exhibits superior biocompatibility in this more inflammation-prone anatomic site, which is not surprising given that immune privilege dampens the foreign body response (Hernandez et al., 2021). Furthermore, our work suggests that these findings are generalizable to other non-immune privileged locations in the body.

The mechanism behind this apparent diminished foreign body response is still unclear, especially in comparison to PDMS, and the hypotheses made here are speculative. According to the literature, stiffness is correlated with more fibrosis via increased biomaterial-tissue mechanical mismatch (Blakney et al., 2012; Previtera and Sengupta, 2015; Jansen et al., 2018; Sridharan et al., 2019; Noskovicova et al., 2021b; Goswami et al., 2021), whereas surface roughness with characteristic lengths similar to the size of cells (Vassey et al., 2020; Doloff et al., 2021) and hydrophilicity (Hotchkiss et al., 2016; Lv et al., 2018; Visalakshan et al., 2019) are associated with beneficial anti-inflammatory macrophage phenotypes. Hence, the observation that SIBS results in less fibrous encapsulation could perhaps be explained by its significantly higher surface roughness and lower contact angle. However, it is unlikely that the small, but significant, differences in physicochemical properties between the two biomaterials are responsible for this, as the effect of these is only really significant with larger differences in the Pa-to-kPa range for stiffness (Blakney et al., 2012; Previtera and Sengupta, 2015; Jansen et al., 2018; Sridharan et al., 2019; Noskovicova et al., 2021b; Goswami et al., 2021), at the microscale for roughness (Vassey et al., 2020; Doloff et al., 2021) and across the defined hydrophobicity-hydrophilicity limit for wettability (Hotchkiss et al., 2016; Lv et al., 2018; Visalakshan et al., 2019). Instead, we believe it is the phase separation that is inherent to block copolymers that drives our findings through differential protein adsorption, as SIBS displays a phase-separated surface morphology (Pinchuk et al., 2008; Ranade et al., 2005; Shen et al., 2021) and this greatly influences protein adsorption (Bhushan and Schricker, 2014; Firkowska-Boden et al., 2018). Perhaps, the hard polystyrene and soft polyisobutylene phases of SIBS preferentially adsorb a particular set of proteins that polarize slightly more macrophages into a pro-inflammatory phenotype with enhanced *TNF* and *CXCL8* expression as well as decreased *TGFB1* expression. This, in turn, could stimulate fibroblasts to shift away from a contractile phenotype to an inflammatory one (Davidson et al., 2021; Schuster et al., 2021) with increased expression of *IL6* and *CXCL8* as well as decreased contractility (Goldberg et al., 2007; Zhu et al., 2021; Iocono et al., 2000; Ghazarian et al., 2000). Given the reduced amount of fibroblasts within the SIBS fibrous capsules and the fact that all capsules are well-formed at 28 days, we think that – akin to the resolution phase of wound healing (Darby et al., 2014; Hinz and Lagares, 2020) – apoptosis is triggered earlier for the SIBS implants. Indeed, lack of sustained TGF-β secretion by macrophages could make fibroblasts sensitive to apoptosis (Zhang and Phan, 1999; Kulasekaran et al., 2009), which would also be induced by the continuous presence of inflammatory cytokines such as TNF-α (Hinz and Lagares, 2020; Wajant et al., 2003) around SIBS implants. As a whole, we speculate that the phase-separated surface morphology of SIBS may promote differential protein adsorption and consequent establishment of an inflammatory pro-apoptotic microenvironment that drives rapid resolution of the foreign body response around SIBS compared to PDMS. Consequently, protein adsorption studies and apoptosis assays are needed to test this hypothesis in the future, as other mechanisms – involving T lymphocytes for example (Doloff et al., 2021; Lin et al., 2025; Yang et al., 2023) – could very well be at play.

### SIBS as a potential biomaterial alternative to PDMS

Considering the generalizable observation that SIBS is more biocompatible with a milder foreign body response, it becomes reasonable to think that its use as an alternative to PDMS could potentially reduce the complications of silicone-based implantable medical devices. Although this remains to be tested, SIBS might reduce the rates of capsular contracture with breast implants and mechanical obstruction of hydrocephalus shunts as well as help preserve residual acoustic hearing after cochlear implant surgery and extend the lifespan of subdermal contraceptive implants, which could perhaps ultimately reduce implant-related morbidity and associated healthcare costs.

Along with the conceivable benefit of a diminished foreign body response, the use of SIBS also presents other advantages and potential opportunities (Table 3). SIBS is hypothesized to be exceptionally resistant to oxidation, hydrolysis and enzymatic degradation (Pinchuk et al., 2008), and – given that silicone-based implantable medical devices shed particles (Dijkman et al., 2021; de Bakker et al., 2023) – it could potentially prevent the latter, further improving its biocompatibility. The remaining advantages of SIBS derive from its block copolymer nature, which confers to it better control of physicochemical properties and protein adsorption as well as thermo- and solution-forming capabilities. Indeed, its mechanical properties (Pinchuk et al., 2008; Shen et al., 2021; Fittipaldi and Grace, 2017) and its protein adsorption dynamics (Bhushan and Schricker, 2014; Firkowska-Boden et al., 2018) can be tuned by altering the composition of its blocks. In addition, thermo-forming capacity enables injection and compression molding (Pinchuk et al., 2008) as well as 3D printing (Shen et al., 2021), while solution-forming makes solvent casting and bonding (Pinchuk et al., 2008) possible as well as dip (Wang et al., 2010) and spray coating (Yuan et al., 2016b). Besides these material processing advantages, SIBS can also be functionalized to carry sulfate (Zhu et al., 2012), nuclease (Yuan et al., 2014b), benzyl chloride (Yuan et al., 2016a), hyaluronic acid (Yuan et al., 2014a) or chitosan (Yuan et al., 2014a) groups as well as reinforced with carbon (Puskas et al., 2010; Rezvova et al., 2022) and infused with lubricating liquids (Yuan et al., 2016b; Yuan et al., 2015) to improve its hemocompatibility (Yuan et al., 2016b; Zhu et al., 2012; Yuan et al., 2015) and mechanical properties (Rezvova et al., 2022) as well as confer to it bactericidal (Yuan et al., 2016a; Yuan et al., 2016b; Yuan et al., 2014b; Yuan et al., 2015) and anti-fouling (Yuan et al., 2014a; Yuan et al., 2016b; Yuan et al., 2015) functions. That being said, silicone can also be modified to significantly improve its biocompatibility by casting it in texturized molds allowing surface roughness modulation (Doloff et al., 2021) as well as functionalizing it with collagen (Majd et al., 2015), human serum albumin (Zhou et al., 2024), interleukin-4 (Kim et al., 2021), dexamethasone (Wilk et al., 2016), pravastatin (Liao et al., 2019), the colony stimulating factor 1 receptor inhibitor (Farah et al., 2019) and the Met-Z2-Y12 antifibrotic agent (Karinja et al., 2023). In addition, zwitterionic coatings can also be used to drastically reduce the fibrous encapsulation of silicone with materials like poly (carboxybetaine methacrylate) (Horne et al., 2023), alginate-poly (ethylene imine) (Zhang et al., 2018) and methacryloyloxyethyl phosphorylcholine (Yesilyurt et al., 2017) polymers.

**TABLE 3:** Advantages and disadvantages of SIBS as a potential biomaterial alternative to PDMS.

Criteria	PDMS	SIBS
Biocompatibility including resistance to oxidation, hydrolysis and enzymatic degradation	+	+++
Inherently tunable physicochemical properties as well as protein adsorption	+	+++
Gas permeability enabling sterilization with ethylene oxide	+++	-
Thermo-formable allowing injection and compression molding as well as 3D printing	-	+++
Solution-formable enabling solvent casting and bonding as well as dip and spray coating	-	+++
Availability and affordability with respect to production	+++	+

Nevertheless, SIBS does have its limitations (Table 3). Compared to PDMS, it is not permeable to gas (Pinchuk et al., 2008), rendering ethylene oxide sterilization difficult, and is susceptible to oil absorption, which results in swelling (Fittipaldi and Grace, 2016) and brittleness (Fittipaldi and Grace, 2017). However, SIBS can also be chemically crosslinked at its hard glassy polystyrene blocks with 4-vinylbenzocyclobutene to prevent this (Sheriff et al., 2015). More importantly however, it is still much more expensive to produce SIBS (Pinchuk et al., 2008) compared to PDMS, which limits its availability and its further implementation as a biomaterial.

## Conclusion

Silicone-based implantable medical devices such as breast implants, hydrocephalus shunts and cochlear implants fail over time in approximately 10% of cases due to an excessive foreign body response to silicone. In this work, we evaluate the thermoplastic elastomer poly (styrene-*block*-isobutylene-*block*-styrene) (SIBS) as a potential alternative to polydimethylsiloxane (PDMS) by studying the foreign body response that they elicit. Although we demonstrate that both biomaterials have comparable physicochemical properties, we also observe that SIBS elicits a milder foreign body response characterized by the formation of a thinner, less cellular, fibrous capsule under the skin in mice. Our *in vitro* experiments show that macrophages display an altered phenotype on SIBS and produce conditioned media that reduces fibroblast contractility and enhances inflammatory gene expression. This leads us to hypothesize that the phase-separated surface morphology of SIBS, through differential protein adsorption, establishes an inflammatory pro-apoptotic microenvironment that accelerates the resolution of the foreign body response. Together, our results highlight SIBS as a promising biomaterial alternative to PDMS in hopes of reducing implant-related morbidity associated with silicone devices.

## Data Availability

The raw data supporting the conclusions of this article will be made available by the authors, without undue reservation.
